# Design and Experiment of a Solder Paste Jetting System Driven by a Piezoelectric Stack

**DOI:** 10.3390/mi7070112

**Published:** 2016-06-30

**Authors:** Shoudong Gu, Xiaoyang Jiao, Jianfang Liu, Zhigang Yang, Hai Jiang, Qingqing Lv

**Affiliations:** School of Mechanical Science and Engineering, Jilin University, No. 5988, Renmin Road, Changchun 130025, China; gusd14@mails.jlu.edu.cn (S.G.); jiaoxy10@mails.jlu.edu.cn (X.J.); yzg@jlu.edu.cn (Z.Y.); jianghai15@mails.jlu.edu.cn (H.J.); lvqq13@mails.jlu.edu.cn (Q.L.)

**Keywords:** solder paste, jetting, piezoelectric, ejection behavior

## Abstract

To compensate for the insufficiency and instability of solder paste dispensing and printing that are used in the SMT (Surface Mount Technology) production process, a noncontact solder paste jetting system driven by a piezoelectric stack based on the principle of the nozzle-needle-system is introduced in this paper, in which a miniscule gap exists between the nozzle and needle during the jetting process. Here, the critical jet ejection velocity is discussed through theoretical analysis. The relations between ejection velocity and needle structure, needle velocity, and nozzle diameter were obtained by FLUENT software. Then, the prototype of the solder paste jetting system was fabricated, and the performance was verified by experiments. The effects of the gap between nozzle and needle, the driving voltage, and the nozzle diameter on the jetting performance and droplet diameter were obtained. Solder paste droplets 0.85 mm in diameter were produced when the gap between the nozzle and needle was adjusted to 10 μm, the driving voltage to 80 V, the nozzle diameter to 0.1 mm, and the variation of the droplet diameter was within ±3%.

## 1. Introduction

In the SMT (Surface Mount Technology) production process, the solder paste printing on the circuit board is often the first step of the entire SMT production process; the printing is also one of the most critical and important processes [1]. The solder paste printing process can be divided into three types: printing, dispensing, and jetting, which is the most recently developed process [2]. Printing is only suitable for mass production because of long production and low flexibility. Needle dispensing relies on the dispensing needle guide solder paste point formed on the substrate. The main types are time pressure [3,4], rotary screw [5], and piezoelectricity [6]. Needle dispensing is a useful supplement in Ball Grid Array (BGA) rework, being solder coated, forming 3D graphics, and other aspects of the screen printing. However, the most important feature of this technology is that there is a need for accurate control of the needle height, that solder dispensing is slow, and that dispensing repeat accuracy is poor. As electronic products miniaturize, and with improvements in density and 3D-structures, traditional solder paste dispensing technology has been unable to meet the existing technology market. The appearance of the solder paste jetting system is a major breakthrough in the SMT industry.

Jetting is the process in which fluid is ejected rapidly through a nozzle, using fluid momentum to break free from the nozzle [7,8]. Jetting technology has been widely used in rapid manufacture [9,10], life sciences [11,12,13], electronic fabrication [14,15], and so on due to the fast manufacturing time, high operating flexibility, and low production cost. To use these advantages in small-scale, high-mix electronics solder paste printing, the Swedish company Mycronic developed the first solder paste jetting system [2,16,17]. In this jetting system, the solder paste is acted upon by instant high pressure from the impact of an plunger and then ejected rapidly onto the substrate through a nozzle [18]. This system can make 3D deposits, a complete BGA rework, and replace printing with mass production. A drawback of this system [2] is that the number of certified jettable solder pastes is limited; these are only supported by the machine supplier.

In order to achieve a wider variety of solder paste jetting for 3D circuitry, a solder paste jetting system driven by piezoelectric stack is introduced and experimented in this paper based on the principle of the nozzle-needle-system. Nozzle-needle-systems [19,20,21,22] are widely used to jetting high viscosity adhesives in electronic packaging technology, in which the fluid in the nozzle orifice is forced by a high pressure, induced by the needle’s impact on the nozzle and jetting out to produce a drop. However, for solder paste, which is a liquid-solid two-phase fluid, the solder particles will be crushed and accumulated under the impact force. That will not only destroy the internal components of the solder paste, but also clog the nozzle orifice. Thus, the present study proposes a solder paste jetting system driven by a piezoelectric stack, in which a miniscule gap exists between the nozzle and needle instead of the needle hitting the nozzle directly. Fundamental equations and simulation analysis by FLUENT software (v6.3.26) for the jetting system are presented to express the critical jet ejection velocity. Then, the prototype of the solder paste jetting system was fabricated, and the performance was then verified by experiments.

## 2. Principle of the Jetting System

### 2.1. The Jetting System Structure

**Figure 1:**
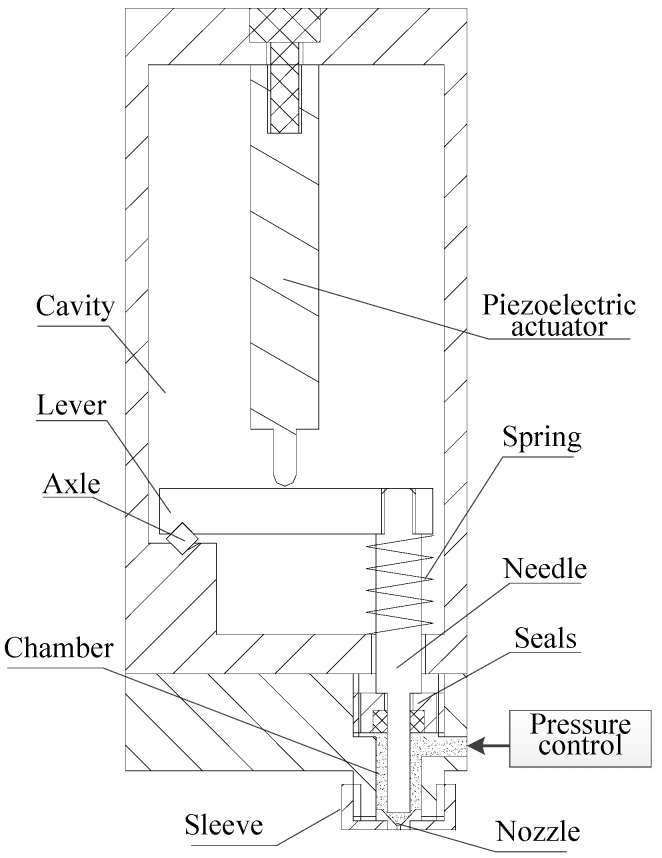
Structure of the solder paste jetting system.

**Figure 2:**
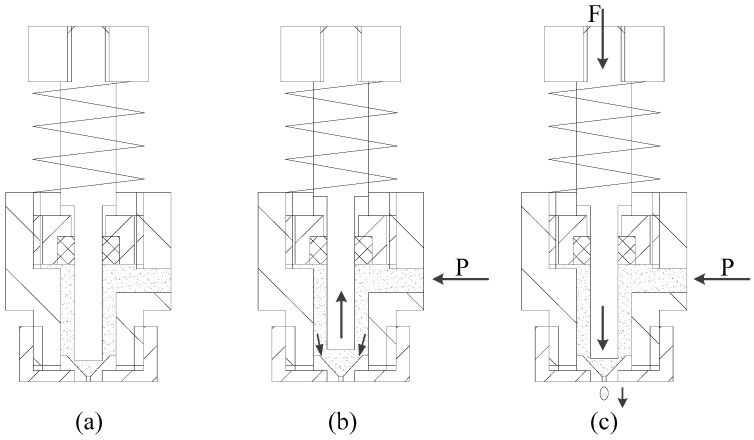
Working principle. (**a**) Normal state; (**b**) Needle upward movement; (**c**) Needle downward movement.

Figure 1 shows the structure of the solder paste jetting system, which consists of the piezoelectric actuator, lever, needle-to-nozzle distance control sleeve, pressure control unit, needle, and nozzle. The difference with the traditional nozzle-needle-system is that a miniscule gap exists between the nozzle and needle instead of the needle hitting the nozzle directly, and it can be adjusted by the sleeve. The principle is shown in Figure 2.

The working principle of the solder paste jetting system is shown in Figure 2.

**Figure 3:**
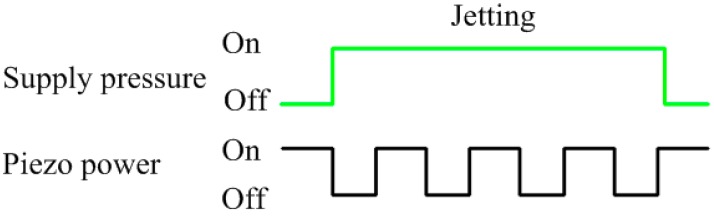
Relation between the supply pressure and piezoelectric power.

1. As shown in Figure 2a, the piezoelectric stack is powered on, and the needle is in suspension under the combined effect of the lever and the force of the spring. A miniscule gap exists between the nozzle and needle. The supply pressure acting on the solder paste is off, and viscous force and surface tension can prevent the solder paste flowing out.

2. As shown in Figure 2b, the needle raises up under the restoring force of the spring when the piezoelectric stack is powered off. At the same time, the supply pressure acting on the solder paste is activated. The relation between the supply pressure and piezoelectric power shows in Figure 3.

3. The needle moves down when an electric field is applied to the piezoelectric stack. The solder paste at the nozzle is forced by the inertial force pressured by the needle to remove the tensile viscous force and surface tension and then jets out from the nozzle orifice so as to produce a drop. The needle does not touch the nozzle during the solder paste jetting process (Figure 2c).

### 2.2. Theoretical Analysis of Jetting

The solder paste at the nozzle is forced by the inertial force pressured by the needle, the tensile viscous force, and surface tension. The tensile viscous force $F_{\eta }$ can be expressed as

(1) $$F_{\eta }=P_{n}\cdot S=\frac{1}{4}\pi d^{2}\cdot P_{n}$$

where *d* is the nozzle diameter, and $P_{n}$ is the tensile stress, which can be expressed as

(2) $$P_{n}=\eta_{n}{\overset{\cdot }{e}}_{n}$$

where $\eta_{n}$ is the extensional viscosity. ${\overset{\cdot }{e}}_{n}$ is the strain rate, which can be expressed as

(3) $${\overset{\cdot }{e}}_{n}=\frac{\nu }{L}$$

where *L* is the length of the droplet, and *v* is the droplet velocity.

Thus, the tensile viscous force $F_{\eta }$ can be expressed as

(4) $$F_{\eta }=\frac{1}{4}\frac{\nu \eta_{n}}{L}\pi d^{2}$$

We assume that the diameter of the solder paste droplet departed from the nozzle is the nozzle diameter *d*. The inertia force generated by the impact of the needle can be expressed as

(5) $$F_{i}=\frac{\pi }{8}\rho v^{2}dL$$

where *ρ* is the solder paste density. Thus, the necessary condition to eject the fluid is

(6) $$F_{i}>F_{\eta }+F_{b}$$

where $F_{b}$ is the surface tension.

As the solder paste jets through a small nozzle orifice, the Weber number can be calculated by

(7) $$We=\frac{\rho \nu^{2}d}{\sigma }$$

where *σ* is surface tension coefficient. It is approximately 0.49 N/m.

When the nozzle diameter *d* is 0.1 mm, the solder paste density *ρ* = 7400 kg/m^3^, the droplet velocity *v* = 1.5 m/s, and the *We* = 3.39 > 1. Thus, the effect of surface tension can be ignored.

(8) $$\frac{F_{i}}{F_{\eta }+F_{b}}\approx \frac{F_{i}}{F_{\eta }}>1\Rightarrow \nu >\frac{2\eta_{n}d}{\rho L^{2}}$$

From (8), in order to achieve the injection of the solder paste, it must ensure that the solder paste velocity exceeds a critical value *v*.

Assume the pressure pressed on the solder paste in the nozzle orifice during the impact process as *F*, viscous resistance at the nozzle orifice as *T*. Under the action of the two forces, solder paste began to flow down, according to the Newton's second law:

(9) $$F-T=ma=\rho V\frac{dv_{\left(t\right)}}{dt}$$

where V is the volume of solder paste.

Viscous resistance T can be expressed as

(10) T=Aτ

where *A* is the area of the solder paste contact with the wall of nozzle orifice, and τ is the shear stress.

However, the solder paste is Bingham plastic. The shear stress τ can be expressed as

(11) $$\tau =(\tau_{0}+2\eta \frac{v_{\left(t\right)}}{d})$$

where *τ*_0_ is shear yield stress, and *η* is the solder paste viscosity.

Based on (9)–(11), the following formula can be obtained:

(12) $$\rho V\frac{dv_{\left(t\right)}}{dt}+\frac{2A\eta }{d}v_{\left(t\right)}=F-A\tau_{0}$$

At the initial state, the solder paste is static, e.g., *υ*_(*t*)_ = 0. Therefore, the solder paste velocity calculated from (12) can be expressed as

(13) $$v_{\left(t\right)}=\frac{F-A\tau_{0}}{2A\eta }d\left(1-e^{-\frac{2A\eta }{\rho Vd}t}\right)$$

From (13), the solder paste velocity is critically influenced by the pressure pressed on the solder paste *F* and the nozzle diameter *d*.

### 2.3. Flow Field Simulation

**Figure 4:**
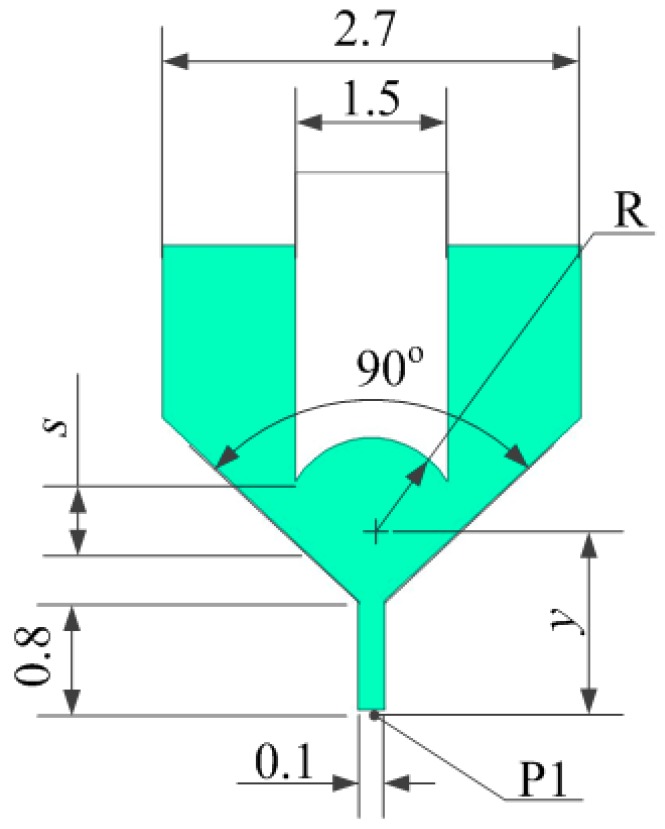
Physical model of the nozzle and needle.

The model structure shown in Figure 4 uses a concave spherical needle to strengthen the driving force by focusing on the solder paste. The simulation model divided the grid and then imported it into FLUENT for analysis by using the mixture model, in which the boundary conditions for the inlet pressure and outlet pressure were 0.2 and 0 MPa, respectively. The solder paste flow could be approximated as a stable laminar flow motion. Certain parameters of the solder paste are shown in Table 1.

#### 2.3.1. Needle Structure

**Figure 5:**
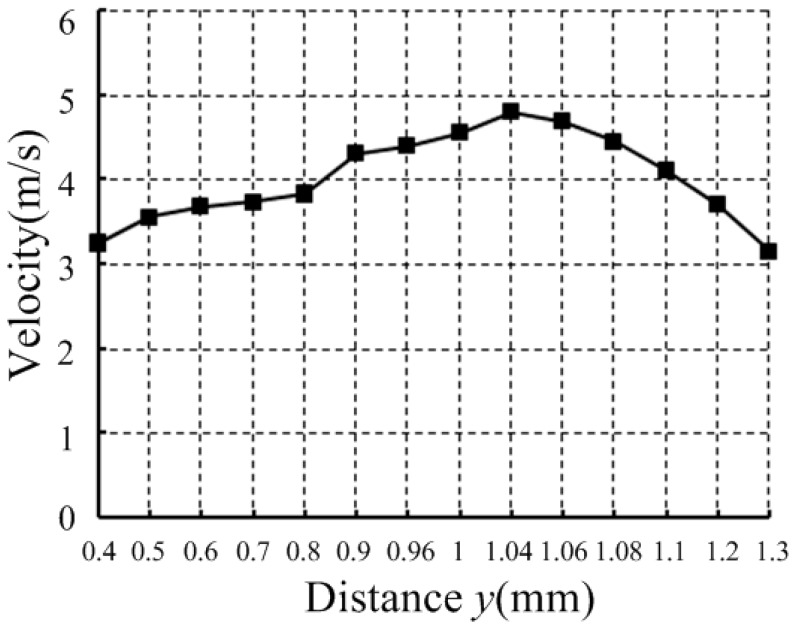
Velocity inside the nozzle orifice changes with distance *y*.

To obtain a suitable concave spherical, we set the different distance *y* between the concave spherical center and the nozzle outlet to simulation, and the different distance *y* corresponds to the different radius *R* according to the physical model when the initial distance *s* between the needle and the nozzle is 200 μm. It can be calculate by $R=\sqrt{{\left(1.7-y\right)}^{2}+0.75^{2}}$. The needle accelerates to move 150 μm within 0.1 ms from a stationary state. When the needle reaches the maximum displacement, the distance *s* between the needle and the nozzle is 50 μm. Figure 5 shows that the solder paste velocity inside the nozzle orifice changes with the distance *y* when the needle moves 150 μm.

Figure 5 shows that, when the distance *y* is 1.04 mm, the solder paste velocity inside the nozzle orifice is at maximum. When the distance is less than 1.04 mm, the solder paste velocity increases. In addition, when the distance is larger than 1.04 mm, solder paste velocity decreases. Thus, when the concave spherical radius *R* = 1 mm, the solder paste velocity inside the nozzle orifice is generated great to jet.

#### 2.3.2. Needle Velocity

**Figure 6:**
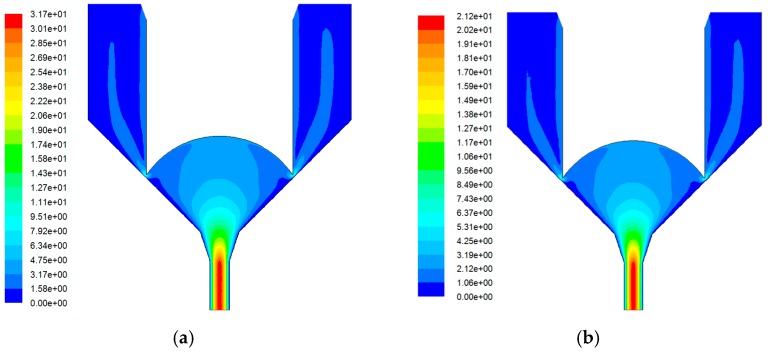

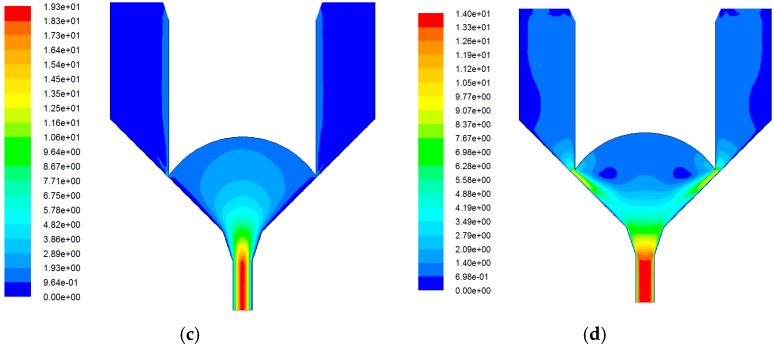
Velocity distribution with different needle velocity. (**a**) *t* = 0.1 ms; (**b**) *t* = 0.15 ms; (**c**) *t* = 0.2 ms; (**d**) *t* = 0.25 ms.

Considering the above simulation boundary conditions, and the needle accelerates to move 180 μm within different times from a stationary state, Figure 6 shows the velocity distribution in the nozzle.

The simulation in Figure 6 indicates that the maximum solder paste velocity in the nozzle orifice is gradually reduced with the increase in the moving time. When the moving times change from 0.1 ms to 0.25 ms, the needle impact velocity dropped from 3 to 1.44 m·s^−1^, and the maximum solder paste velocity drops from 31.7 to 14 m·s^−1^. One reason is that the solder paste jetting relies on the pressure in the chamber to instantly increase in a short time by the movement needle. High needle velocity easily produces high instantaneous pressure in the gap between nozzle and needle, which also corresponds to higher ejection velocity. Predictably, solder paste injection cannot be formed when the movement time of the needle is greater than a certain value.

#### 2.3.3. Nozzle Diameter

**Figure 7:**
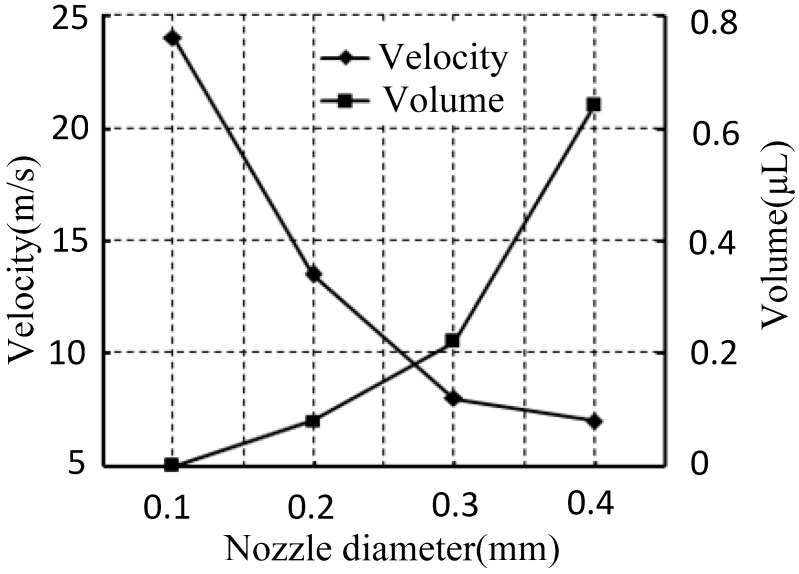
Velocity and volume changes with nozzle diameter.

The nozzle diameter is also one of the key factors that affect solder paste injection velocity. With the above simulation boundary conditions and the needle accelerating to move 180 μm within 0.1 ms from a stationary state, the velocity at P1 can be obtained at different nozzle diameters. Then, according to Equation (14), the paste droplet volume can be calculated at different nozzle diameters, as shown in Figure 7:

(14) $$Q=\int_{t_{b}}^{t_{c}}v_{\left(t\right)}\pi r^{2}dt$$

where $t_{b}$ is the needle movement’s start time (s), and $t_{c}$ is the needle movement’s end time (s).

Figure 7 shows that, with the smaller nozzle diameter, the paste droplet volume jetting is smaller, and the velocity in the nozzle orifice is bigger. That is, the resistance is smaller in the bigger nozzle diameter when the needle moves down. The solder paste in a bigger nozzle orifice is easier to flow with a smaller pressure. Thus, the pressure in the gap between the nozzle and needle will be reduced with the increase in the nozzle diameter. Then, the jetting velocity generated will be reduced by a small amount of pressure. Therefore, the volume of injection solder paste can also be adjusted by choosing different nozzle orifice diameters.

## 3. Experiment

### 3.1. Experimental Set-Up

**Figure 8:**
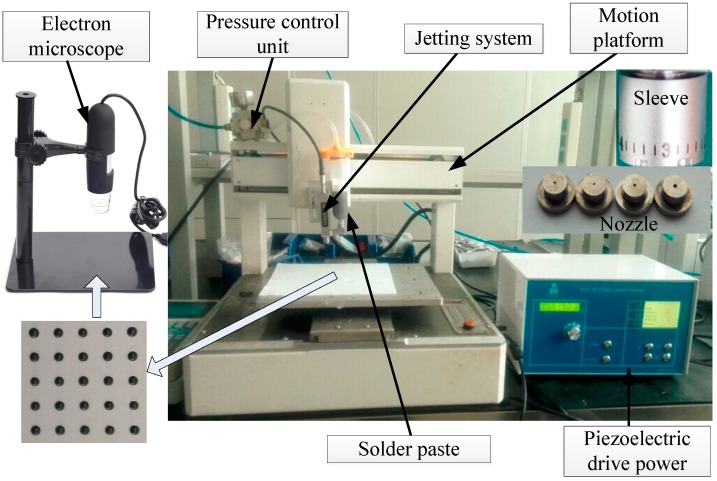
Experiment test system of the solder paste jetting system.

The prototype of the solder paste jetting system was fabricated following the simulation model. The experimental system was comprised of the solder paste jetting system, piezoelectric drive power, a precision electron microscope, a motion platform, and a pressure control unit, as shown in Figure 8.

The piezoelectric drive power can offer a precisely voltage signal to the piezoelectric stack. The pressure control unit can switch the supply pressure. When the jetting system jets solder paste drops, the supply pressure is on. The solder paste used in the experiments is SMIC M705-GRN360-K2-VT. The main ingredient is Sn96.5/Ag3.0/Cu0.5, and the particle diameter is 25–36 μm. Its density and viscosity is usually 7400 kg/m^3^ and 180–220 Pa·s. The experimental study of the solder paste jetting system was carried out under different conditions, including the gap between the nozzle and needle, the driving voltage amplitude, and the nozzle diameter. The electron microscope was used to measure the diameter of each droplet.

### 3.2. Gap Between Nozzle and Needle

**Figure 9:**
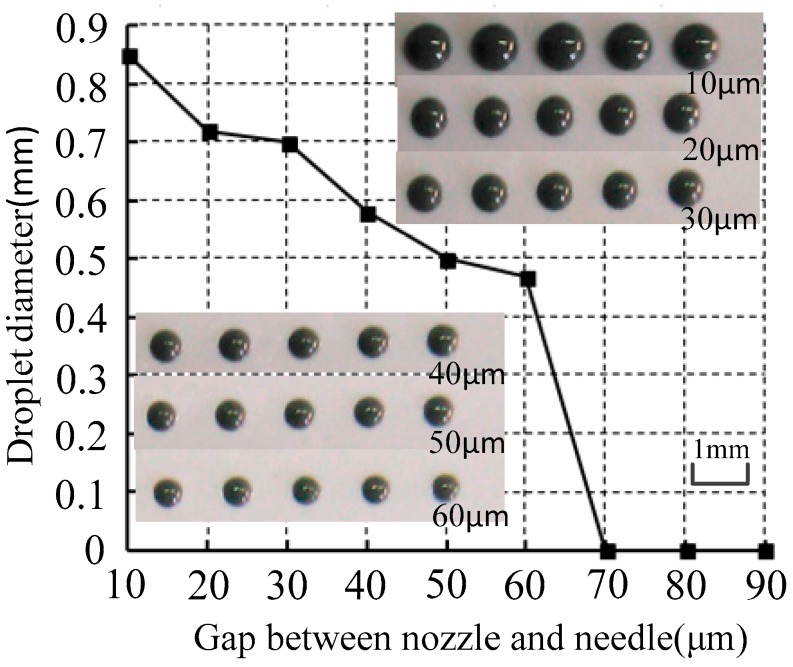
Droplet diameter changes with the gap between nozzle and needle.

The gap was adjusted by the sleeve. The piezoelectric drive power output voltage (80 V) was set so that the needle reached maximum displacement. We adjusted the sleeve and nozzle upward until the nozzle contacted with the needle, and we adjusted then the sleeve and nozzle downward. Scales were present on the sleeve, and the nozzle moved 10 μm relative to the needle of each scale. In the experiment, the nozzle diameter, supply pressure, and drive signal were 0.1 mm, 0.6 MPa, and high-voltage 80 V, 5 ms; low-voltage 0 V, 2.5 ms, respectively. The droplet diameter under different gap between the nozzle and needle is shown in Figure 9.

**Figure 10:**
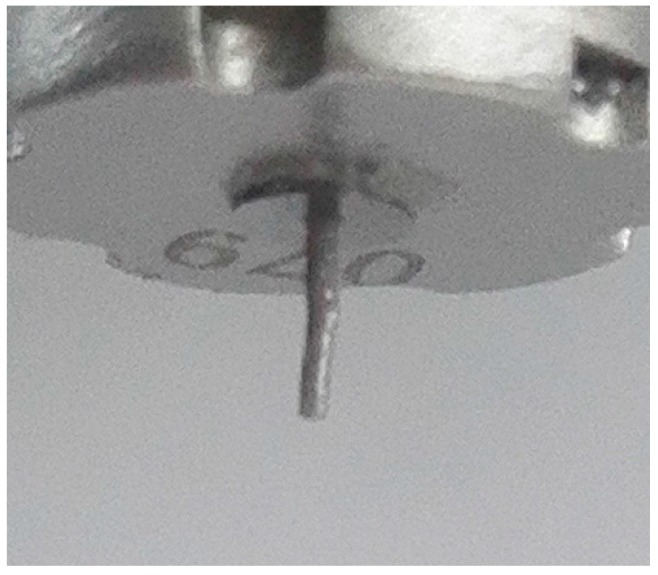
Solder paste suspension at the nozzle.

As shown in Figure 9, the droplet diameter was inversely proportional to the gap. The smaller the gap was, the bigger the droplet diameter of injected solder paste became. However, when the gap was bigger than 60 μm, the solder paste could not jet out and instead hung at the nozzle, as shown in Figure 10. That is, the bigger the gap between the nozzle and needle was, the smaller the instantaneous pressure generated between the nozzle and needle when the needle moved toward the nozzle. So that, the smaller amount of solder paste can be injected. When the gap increased to more than 60 μm, the instantaneous pressure was not sufficient to generate enough velocity to overcome the tensile viscous force, and the solder paste could not be separated from the nozzle. Assuming the solder paste viscosity *η* is constant, so that the extensional viscosity $\eta_{n}$ was three times that of the solder paste viscosity *η*. From Equation (8), the ejection velocity to achieve injection had to exceed 1.54 m/s when the gap was 60 μm.

### 3.3. Driving Voltage

**Figure 11:**
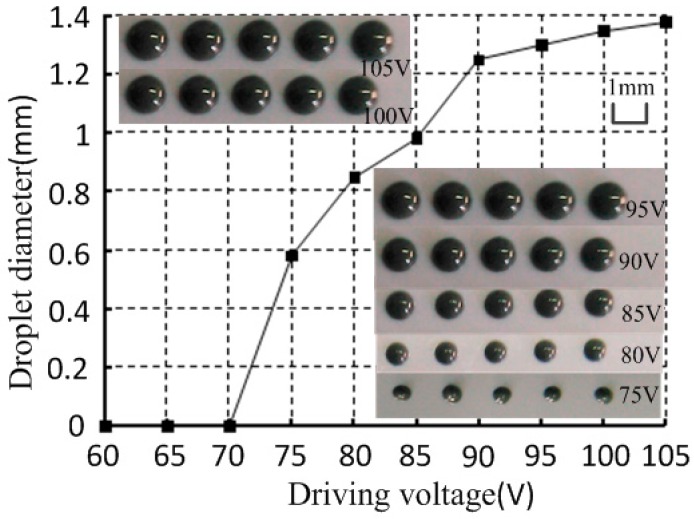
Relationship between driving voltage and droplet diameter.

With a supply pressure of 0.6 MPa, and a nozzle diameter of 0.1 mm, the gap between the nozzle and needle was 10 μm with different driving voltages (high voltage 5 ms, low voltage 2.5 ms, 0 V). As shown in Figure 11, the droplet diameter and the driving voltage was directly proportional when the driving voltage was adjusted from 70 to 105 V. However, when the driving voltage decreased below 70 V, the solder paste could not jet out and instead hung at the nozzle. This phenomenon was mainly because the driving voltage could control the needle motion displacement. When the needle motion displacement increased, the solder paste velocity in the nozzle orifice would increase, and the amount of solder paste that could be injected would increase under such conditions. However, when the needle motion displacement decreased to a point that was smaller than a certain value, the solder paste in the nozzle orifice could not obtain enough velocity to separate from the nozzle. Thus, a higher driving voltage is needed to generate an enough velocity to achieve the solder paste jetting.

### 3.4. Nozzle Diameter

**Figure 12:**
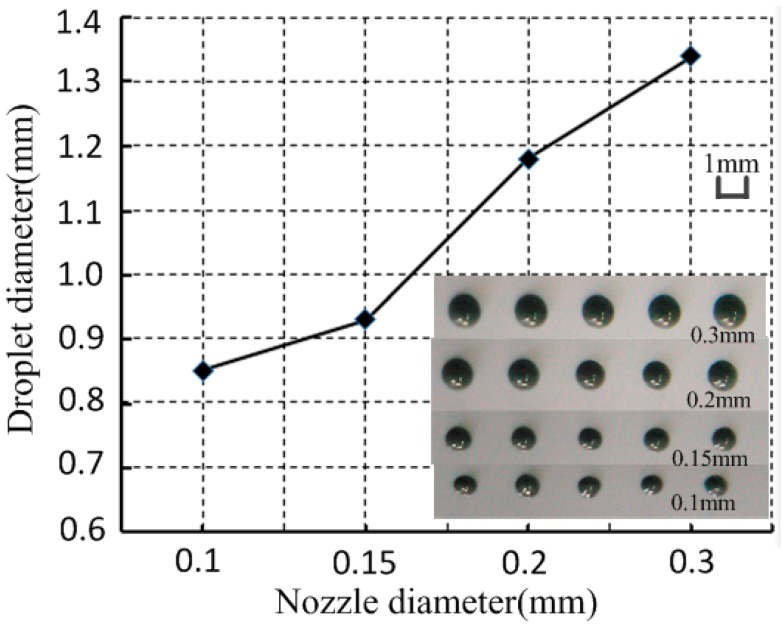
Relationship between nozzle diameter and droplet diameter.

With the supply pressure, the gap between the nozzle and needle and the drive signal were 0.6 MPa, 10 μm, and high-voltage 80 V, 5 ms; low-voltage 0 V, 2.5 ms, respectively. The experiment conducted under different nozzle diameters, and the relationship between nozzle diameter and droplet diameter, is shown in Figure 12.

Figure 12 shows that the solder paste droplet diameter decreased as the nozzle diameter decreased. From the previous simulation analysis, we know that the larger the nozzle orifice is, the more solder paste will be injected out from the nozzle. However, the solder particles were more easily crushed and accumulated under the impact force with a smaller nozzle orifice. Thus, the nozzle orifice used to injection solder paste is usually 0.1 mm or more.

### 3.5. Consistency Analysis

**Figure 13:**
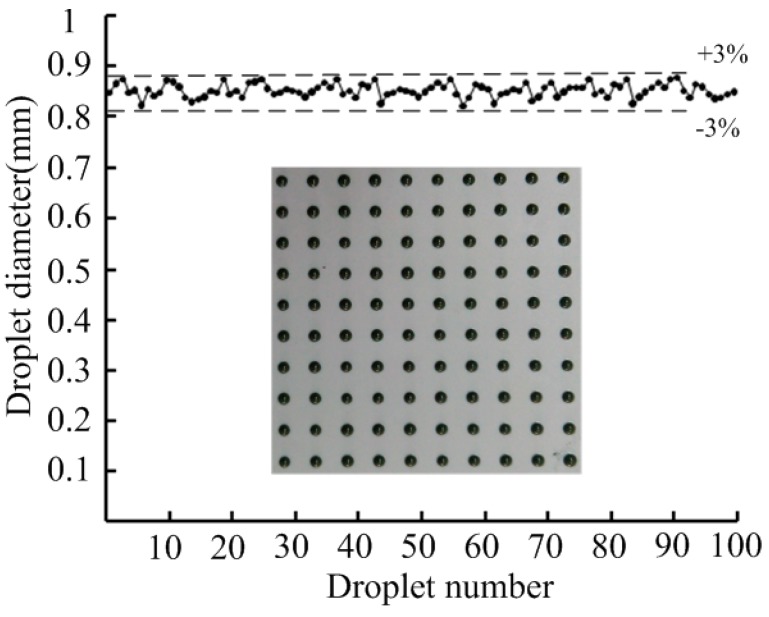
Solder paste droplet diameter distribution.

The same solder paste was used in the experiment. Multiple droplets on the same location or droplets ejected quickly were achieved by the solder paste jetting system. Figure 13 shows a 10 × 10 droplet array with an average diameter of 0.85 mm when the gap between the nozzle and needle was adjusted to 10 μm, the driving voltage to 80 V, the nozzle diameter to 0.1 mm, and the variation of the droplet diameter was within ±3%.

## 4. Conclusions

The solder paste jetting system based on the principle of a nozzle-needle-system, in which a small gap exists between the nozzle and needle during the jetting process, was newly devised here. This system consists of the piezoelectric actuator, a lever, a needle-to-nozzle distance control sleeve, a pressure control unit, a needle, and a nozzle. The critical jet ejection velocity is discussed through theoretical analysis. The relations between ejection velocity and needle structure, needle velocity and nozzle diameter were obtained with FLUENT software. This analysis provides a guideline in the design step before fabricating the jetting system, whose structure can more easily jet solder paste. The prototype of the solder paste jetting system was fabricated and the experiment test system was then designed. The effects of the gap between the nozzle and needle, the driving voltage, and the nozzle diameter on the jetting performance and droplet diameter were obtained. The droplet diameter increased as the driving voltage or the nozzle orifice diameter increased, and as well as the gap between the nozzle and needle decreased. Therefore, the droplet diameter could be controlled by changing the gap between the nozzle and needle, nozzle orifice diameter, and the driving voltage. Furthermore, solder paste droplets with a diameter of 0.85 mm were produced when the gap between the nozzle and needle was adjusted to 10 μm, the driving voltage to 80 V, the nozzle diameter to 0.1 mm, and the variation of the droplet diameter was within ±3%.

## Figures and Tables

**Table 1:** Parameters of the solder paste.

Liquid Density *ρ* (kg/m^−3^)	Viscosity *μ* (Pa·s)	Solid Density (kg/m^−3^)	Particle Diameter (μm)
1225	40	8600	20
